# Novel Transmembrane Receptor Involved in Phagosome Transport of Lysozymes and β-Hexosaminidase in the Enteric Protozoan *Entamoeba histolytica*

**DOI:** 10.1371/journal.ppat.1002539

**Published:** 2012-02-23

**Authors:** Atsushi Furukawa, Kumiko Nakada-Tsukui, Tomoyoshi Nozaki

**Affiliations:** 1 Department of Parasitology, National Institute of Infectious Diseases, Toyama, Shinjuku-ku, Tokyo, Japan; 2 Department of Parasitology, Gunma University Graduate School of Medicine, Showa-machi, Maebashi, Japan; 3 Graduate School of Life and Environmental Sciences, University of Tsukuba, Tennoudai, Tsukuba, Ibaraki, Japan; University of California Los Angeles, United States of America

## Abstract

Lysozymes and hexosaminidases are ubiquitous hydrolases in bacteria and eukaryotes. In phagocytic lower eukaryotes and professional phagocytes from higher eukaryotes, they are involved in the degradation of ingested bacteria in phagosomes. In *Entamoeba histolytica*, which is the intestinal protozoan parasite that causes amoebiasis, phagocytosis plays a pivotal role in the nutrient acquisition and the evasion from the host defense systems. While the content of phagosomes and biochemical and physiological roles of the major phagosomal proteins have been established in *E. histolytica*, the mechanisms of trafficking of these phagosomal proteins, in general, remain largely unknown. In this study, we identified and characterized for the first time the putative receptor/carrier involved in the transport of the above-mentioned hydrolases to phagosomes. We have shown that the receptor, designated as cysteine protease binding protein family 8 (CPBF8), is localized in lysosomes and mediates transport of lysozymes and β-hexosaminidase α-subunit to phagosomes when the amoeba ingests mammalian cells or Gram-positive bacillus *Clostridium perfringens*. We have also shown that the binding of CPBF8 to the cargos is mediated by the serine-rich domain, more specifically three serine residues of the domain, which likely contains trifluoroacetic acid-sensitive O-phosphodiester-linked glycan modifications, of CPBF8. We further showed that the repression of CPBF8 by gene silencing reduced the lysozyme and β-hexosaminidase activity in phagosomes and delayed the degradation of *C. perfringens*. Repression of CPBF8 also resulted in decrease in the cytopathy against the mammalian cells, suggesting that CPBF8 may also be involved in, besides the degradation of ingested bacteria, the pathogenesis against the mammalian hosts. This work represents the first case of the identification of a transport receptor of hydrolytic enzymes responsible for the degradation of microorganisms in phagosomes.

## Author Summary

Phagocytosis is the cellular process of engulfing solid particles to form an internal phagosome in protozoa, algae, and professional phagocytes of multicellular eukaryotic organisms. In phagocytic protozoa, phagocytosis is involved in the acquisition of nutrients, and the evasion from the host immune system and inflammation. While hydrolytic enzymes that are essential for the efficient and regulated degradation of phagocytosed particles, such as bacteria, fungi, and eukaryotic organisms, have been characterized, the mechanisms of the transport of these proteins are poorly understood. In the present study, we have demonstrated, for the first time, the molecular mechanisms of how the digestive enzymes are transported to phagosomes. Understanding of such mechanisms of the transport of phagosomal proteins at the molecular level may lead to the identification of a novel target for the development of new preventive measures against parasitic infections caused by phagocytic protozoa.

## Introduction

Lysozymes (EC 3.2.1.17) are the antibacterial protein that has an ability to damage the cell wall of bacteria [1]. The enzyme acts by catalyzing the hydrolysis of 1,4-beta-linkages between *N*-acetylmuramic acid and *N*-acetyl-D-glucosamine in peptidoglycans and between the *N*-acetyl-D-glucosamine residues in chitodextrins. While biochemical [2], functional [3], and structural [4] features of lysozymes have been well established, the mechanisms for intracellular trafficking and secretion remain poorly characterized except for the report that showed that condroitin sulfate is involved in lysosomal targeting of lysozymes [5]. Hexosaminidase (EC 3.2.1.52) is involved in the hydrolysis of terminal N-acetyl-D-hexosamine residues in hexosaminides. Three dimeric isozymes of β-hexosaminidase are formed by the combination of α and β subunits, encoded by *HEXA* and *HEXB* genes, respectively. β-Hexosaminidase and the cofactor GM2 activator protein catalyze the degradation of the GM2 gangliosides containing terminal N-acetyl hexosamines [6]. Mutations in *HEXA* gene decrease the hydrolysis of GM2 gangliosides, which is the main cause of Tay-Sachs disease, whereas mutations in *HEXB* gene results in Sandhoff disease [7]. The trafficking mechanism of β-hexosaminidase via mannose-6-phosphate receptor has been well studied in mouse lymphoma and myeloma cell [8]–[10]. However, the mechanisms of trafficking of β-hexosaminidase in eukaryotes besides mammals remain to be discovered.

Lysozyme and β-hexosaminidase are abundant components found in phagosomes from *Entamoeba histolytica* [11], [12], which is the anaerobic or microaerophilic protozoan parasite, causing amebic dysentery and amebic liver abscesses in an estimated 10 million cases annually [13]. However, the role and intracellular trafficking of these enzymes remain unknown. Phagocytosis and phagosome biogenesis seems to play a pivotal role in pathogenesis in *E. histolytica* [14]. *E. histolytica* is capable of internalizing extracellular particles by phagocytosis. The amebic trophozoites ingest microorganisms in the large intestine [15], [16], and host cells including non-immune cells [17], and immune cells [18] during tissue invasion. It has been well-established that in vitro and in vivo virulence correlates well with the ability of phagocytosis [14], [19], [20]. Furthermore, phagosomes contain a panel of proteins that were shown to be crucial in pathogenesis such as cysteine proteases (CPs) [21], amoeba pores [22], and galactose/N-acetylgalactosamine-specific lectin [23], [24], proteins involved in cytoskeletal reorganization [25], [26], vesicular trafficking [27]–[29], and signal transduction [30], [31]. Therefore, understanding the molecular mechanisms of phagocytosis and phagosome biogenesis as well as the role and trafficking of individual phagosomal proteins in phagosomes, should help to understand underlying links between phagocytosis and pathogenicity.

Recently, the proteins and mechanisms involved in phagocytosis have been demonstrated. For instance, the surface Ca^2+^-binding kinase (C2PK) has shown to be involved in the initiation of phagocytosis [31]. The antisense inhibition of C2PK caused inhibition of the initiation of erythrophagocytosis. It has also been shown that surface transmembrane kinase (TMK96) and p21-activated kinase (PAK) play an important role in phagocytosis of human erythrocytes [32], [33]. The unconventional myosin, myosin IB, was shown to be involved in cytoskeleton rearrangement during phagocytosis [25], [26]. Furthermore, phosphatidylinositides also play critical roles during phagocytosis [34], [35]. Our previous proteomic studies, where 159 proteins were identified from purified phagosomes [11], [12], also suggested a direct link between phagosome biogenesis and pathogenesis, as phagosomes contained a panel of proteins that were shown to be crucial in pathogenesis described above. Furthermore, the proteins that are implicated for degradation of phagocytosed bacteria, e.g. amoebapores [22], lysozymes, and β-hexosaminidase, as well as other hydrolytic enzymes such as amylase and ribonuclease were also demonstrated in phagosomes. While both the constituents of phagosomes and the kinetics of their recruitment are known, very little is known on how these proteins are transported to phagosomes. Recently, we discovered a putative transmembrane receptor for cysteine proteases from *E. histolytica*, which preferentially binds to CP5 (Nakada-Tsukui K, et al., unpublished data), which is directly implicated in the pathogenesis [36]–[38]. The *E. histolytica* genome contained a total of 11 members showing significant mutual identity and structural conservation to the transmembrane cysteine protease receptor: the signal peptide at the amino terminus, a single transmembrane domain close to the carboxyl terminus, and the YxxΦ motif at the carboxyl terminus. This family of proteins was designated as cysteine protease binding family proteins 1–11 (CPBF1-11). In the present study, we characterized one of the most highly expressed CPBF genes among the family, *CPBF8*. We showed that CPBF8 localizes to phagosomes during phagocytosis, while it is distributed to the acidic compartment in steady state. Affinity immunoprecipitation followed by LC-MS/MS analysis showed that CPBF8 specifically bound to lysozymes and β-hexosaminidase α-subunit. Repression of CPBF8 by gene silencing reduced lysozyme and β-hexosaminidase activities in phagosomes, and caused a defect of digestion of ingested bacteria.

## Results

### Localization of CPBF8

**Figure 1:**
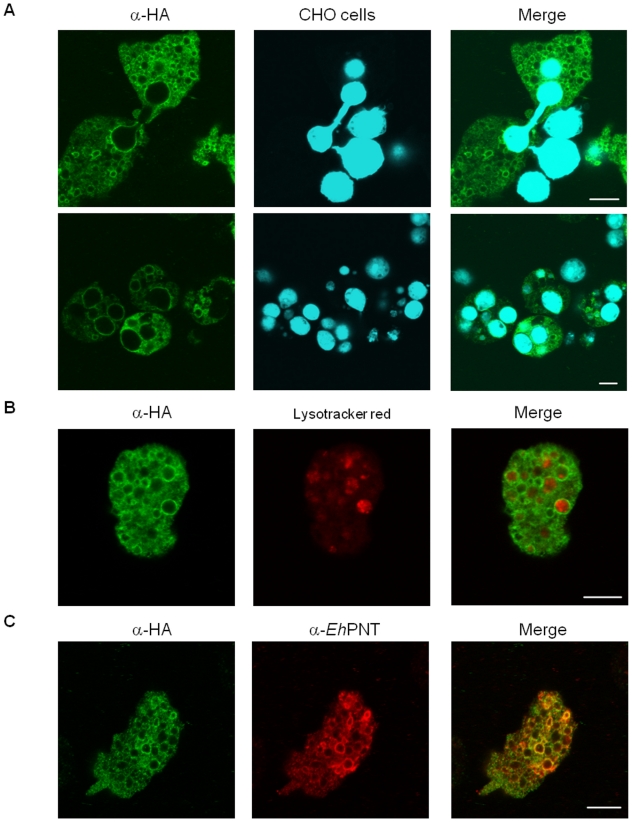
Localization of CPBF8 in *E. histolytica*. (A) Phagosome localization of CPBF8. Amoebae were incubated with Cell Tracker Blue-stained CHO cells (*blue*) for 10 (top row) or 60 minutes (bottom row), fixed, and reacted with anti-HA antibody (*green*). *Bar*, 10 µm. (B) Lysosomes localization of CPBF8. Amoebae were labeled with LysoTracker Red (*red*) and subjected to immunofluorescence assay with anti-HA antibody (*green*). Bar, 10 µm. (C) Colocalization of *Eh*PNT and CPBF8. The cells were fixed, and reacted with anti-*Eh*PNT (*red*) and anti-HA antibody (*green*). Bar, 10 µm.

We examined the localization of CPBF8 during phagocytosis of CHO cells. Trophozoites of CPBF8-HA-expressing strain were incubated with CellTracker-loaded CHO cells for 10 to 60 min to allow ingestion of CHO cells. Immunofluorescence assay using anti-HA antibody showed that CPBF8 was localized to phagosomes containing CHO cells at all time points (10, 30, and 60 mins) (Figure 1A). CPBF8 remained associated with phagosomes in the course of phagocytosis: the percentage of colocalization did not significantly changed (84, 92 and 82% at 10, 30, and 60 min, respectively). Immunofluorescence image of the amoeba undergoing engulfment revealed that CPBF8 localized to the basolateral portion of a phagosome, and excluded from the tunnel-like structure connecting a phagosome and the CHO cell being aspirated [35].

As immunofluorescence assay showed that CPBF8 was also distributed to a large number of vesicles and vacuoles under quiescent (i.e., non-phagocytic) conditions, we examined the nature of these compartments. CPBF8-HA was associated with the acidic organelles labeled with membrane-diffusible LysoTracker under steady-state conditions (60% of LysoTracker-positive vesicles/vacuoles was positive for CPBF8) (Figure 1B). CPBF8 colocalized nicely with a vacuolar membrane protein, pyridine nucleotide transhydrogenase, *Eh*PNT, which converts NADPH and NADH using the proton gradient across the membrane [39] (Figure 1C). It has been shown that *Eh*PNT is localized to the acidic compartment in steady state and transported to phagosomes upon phagocytosis [40].

### CPBF8 binds to β-hexosaminidase α-subunit and lysozymes

**Figure 2:**
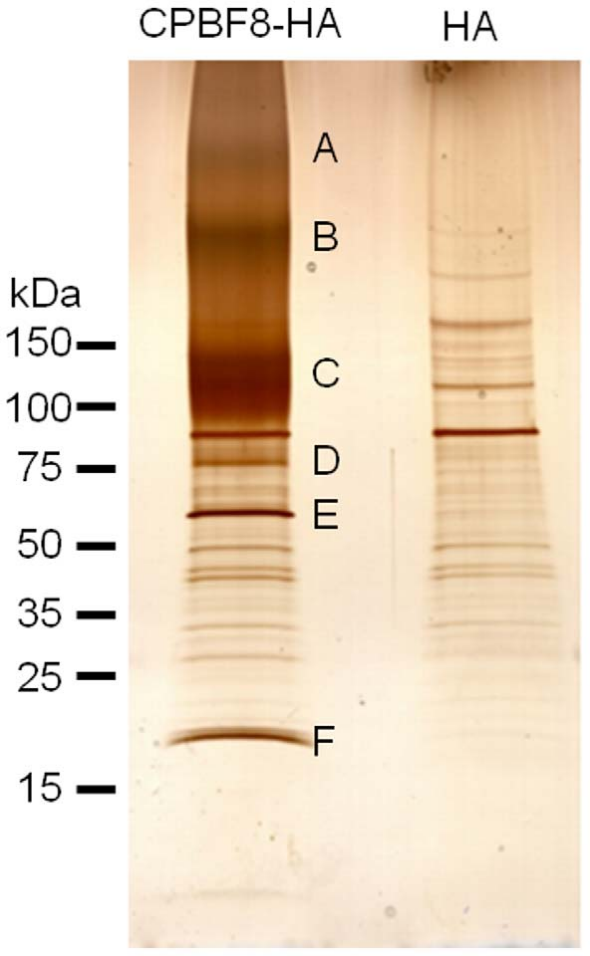
Isolation and identification of CPBF8-binding proteins. Lysates of CPBF8-HA and control (“HA”) transformants were mixed with anti-HA-antibody-conjugated agarose, washed, and eluted with HA peptide. Immunoprecipitated samples were separated on SDS-PAGE and silver stained. Apparent molecular weight of standards (kDa) are indicated on the left. Six bands excised for protein identification are marked (A–F).

To identify potential cargo proteins that CPBF8 binds and carries to phagosomes, we immunoprecipitated proteins that bind to CPBF8, from the lysates of the transformant where HA-tagged CPBF8 was ectopically expressed (Figure 2). Silver stained SDS-PAGE gel revealed three major bands of about >120, 60, and 20 kDa (bands C, E, and F) and three minor bands of about >300, >200, and 75 kDa (bands A, B, and D) exclusively found in the immunoprecipitated sample from CPBF8-HA strain, but not from HA control strain. These bands were excised and subjected to LC-MS/MS analysis (Table 1 and Table S1). Smeary band C, which showed an apparent molecular mass of ∼130 kDa on SDS-PAGE was identified as CPBF8 itself; the apparent size was larger than the predicted size (99.3 kDa), suggestive of post-translational modifications or aberrant structure (see below). Band E was identified as β-hexosaminidase α-subunit (XP_657529; EHI_148130) with 19.7% coverage. Band F was identified as a mixture of lysozyme 1 (XP_653294, EHI_199110) and lysozyme 2 (XP_656933; EHI_096570) with 22.7 and 30.2% coverage, respectively. Lysozyme 1 and 2 were previously demonstrated by our previous phagosome proteome analysis [11], [12]. Bands A, B, and D mostly corresponded to CPBF8 (Table S1). These data clearly indicate that β-hexosaminidase α-subunit and lysozymes are predominant proteins that bind CPBF8. β-hexosaminidase α-subunit was not previously detected by phagosome proteomics, whereas its β-subunit was detected.

**Table 1:** Identification of CPBF-binding proteins by LC-MS/MS analysis.

Size of excised band (kDa) (name)	ID number of identified protein (GenBank ID)	Coverage (%)^1^	Annotation	Predicted molecular weight (kDa)
100–150 (C)	EHI_059830 (XM_647807)	35.1	CPBF8	99.3
60 (E)	EHI_148130 (XM_652437)	19.7	β-hexosaminidase α-subunit	61.0
20 (F)	EHI_199110 (XM_648202)	22.7	lysozyme	23.5
	EHI_096570 (XM_651841)	30.2	lysozyme	23.4

1 coverage based on the peptides over 95% probability.

### Repression of CPBF8 by gene silencing decreases β-hexosaminidase and lysozyme activity

**Figure 3:**
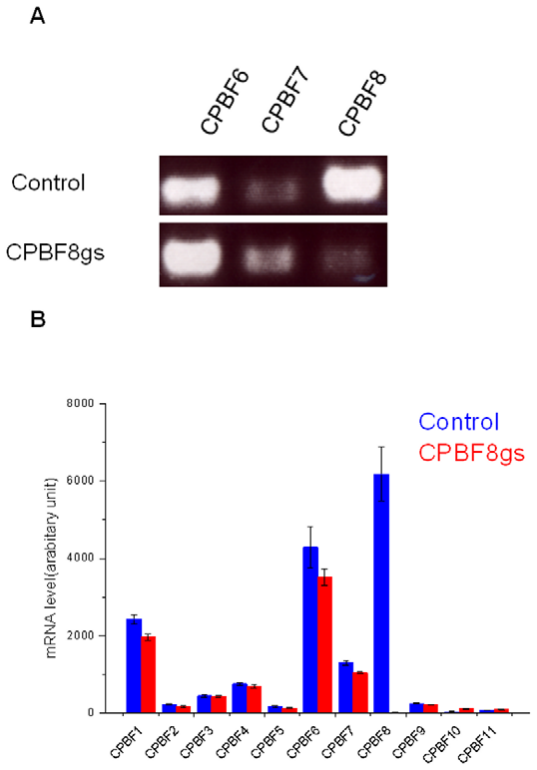
Specific repression of CPBF8 gene in CPBF8gs strain. (A) RT-PCR analysis. A 200 bp long partial *CPBF8* gene was amplified using cDNA from control and CPBF8gs strains. (B) DNA microarray analysis of CPBF genes (CPBF1-11). The raw fluorescence data of triplicates is shown.

To further demonstrate the role of CPBF8, we created a strain in which CPBF8 expession was repressed by long term transcriptional gene silencing [41] (“CPBF8gs strain”). Gene silencing is mediated by nuclear localized antisense small RNAs with 5′-polyphosphate termini [42], and observed only in G3 and its derived strains, in which amoebapore genes have been repressed. RT-PCR analysis showed that the mRNA level of *CPBF8* gene in CPBF8gs strain was specifically reduced to the undetectable level (Figure 3A). DNA microarray analysis further verified that CPBF8 transcript was reduced by 326 fold, while the expression of other CPBF genes remained unchanged (Figure 3B). In *in vitro* cultivation CPBF8gs strain did not show any defect in growth compared to control pSAP2-Gunma-transfected strain (Supplemental information Figure S1). The doubling times of control and CPBF8gs strains were comparable (20.9 and 20.6 h, respectively). Thus, the defects in protein transport and the decrease in cytopathy against mammalian cells and bacteria digestion, described below, are not likely attributable to poor proliferation (growth) of CPBF8gs strain.

**Figure 4:**
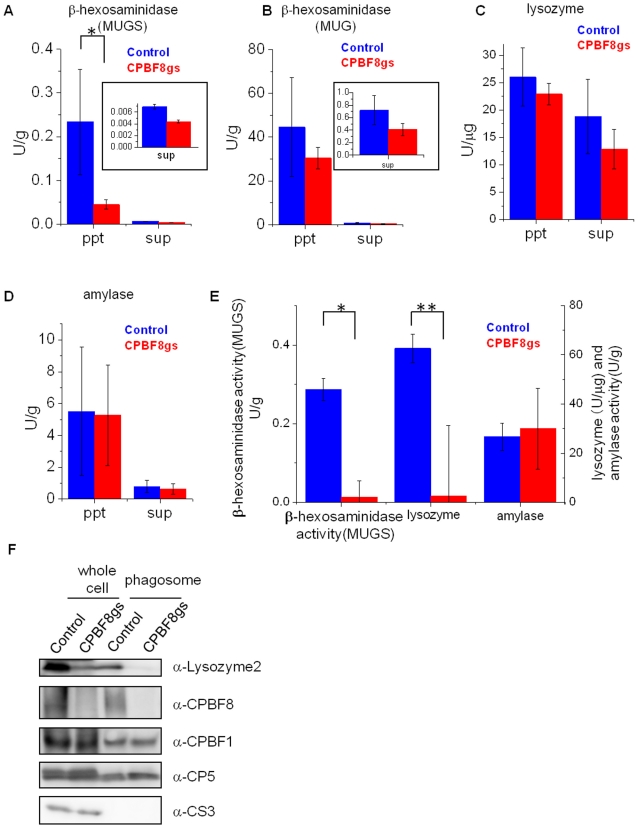
Enzymatic activities in total lysates, culture supernatant, and phagosomes and immuno blot analysis of total lysates and phagosomes derived from control and CPBF8gs strains. After the amoebas were incubated in fresh medium for 2 h, trophozoites and culture supernatant were separated by brief centrifugation. After the supernatant was removed (“sup”), the cell pellet was resuspended and solublized in lysis buffer (“ppt”). To isolate phagosomes (E), the amoebas were incubated with latex beads, and, after brief centrifugation, the cell pellet was resuspended, and mechanically homogenized. The phagosomes were isolated by ultracentrifugation on a sucrose gradient. Enzymatic activities of culture supernatant, whole lysate (A–D), and phagosomes (E) are shown. (A–D) Enzymatic activities of β-hexosaminidase activity toward MUGS (A) and MUG (B), lysozyme activity (C), and amylase activity (D) in the cell pellet and culture supernatant. (E) Enzymatic activities of β-hexosaminidase toward MUGS, lysozyme, and amylase in phagosomes. Data shown are the means ± standard deviations of three independent experiments. “*” or “**” represents statistical significance at p<0.01 or p<0.05, respectively. (F) Immuno blot analysis of the cell pellet and the phagosome fraction. Approximately 20 µg of the cell pellet and 2 µg of the phagosome fraction were electrophoresed. CPBF1 and CP5 are phagosomal proteins, while cysteine synthase 3 (CS3) is a cytosolic marker. Abbreviations are: ppt, pellet fraction; sup, culture supernatant; phagosome, phagosome fraction.

We examined β-hexosaminidase and lysozyme activities in CPBF8gs and control strains using a synthetic N-acetylglucosamine-related substrate (4-methylumbelliferyl-2-acetamido-2-deoxy-β-D-glucopyranoside, MUG) and its sulfo derivative (MUGS) (for β-hexosaminidase), and Bodipy-conjugated *Micrococcus lysodeikticus* cell wall (for lysozymes). The enzyme activity toward MUGS in the whole cells of CPBF8gs strain (0.045 U/g) decreased by 81%, compared to control (0.234) (Figure 4A), whereas that toward MUG reduced by 32% (44.4 and 30.4 U/g in control and CPBF8gs strain, respectively) (Figure 4B). The activity toward MUGS or MUG is known to attributable to β-hexosaminidase activity of a homodimer of α-subunit, or that of both a homodimer of β-subunit and a α/β-subunit heterodimer [43]. The β-hexosaminidase activity toward MUGS and MUG, secreted to the culture medium, also decreased by 37 and 43% in CPBF8gs strain, respectively. The lysozyme activities in the whole cell lysates of CPBF8gs strain appear to be slightly decreased (4.3%), while the amylase activity remained unchanged (Figures 4, C and D). One should know that the degree of lysozyme secretion was much higher than that of β-hexosaminidase. β-Hexosaminidase activity detected in the culture supernatant was almost negligible (Figure 4A and B), and may be attributable to lysed cells. In addition, lysozyme activity detected in the whole lysates and the culture supernatant appear to be attributable to proteins other than lysozyme 1 and 2 because the lysozyme activity in the isolated phagosomes and the amount of lysozyme 2 in the whole cells and phagosomes detected by specific antibody in immunoblot analysis greatly decreased (see below).

### Phagosome targeting of β-hexosaminidase and lysozymes is inhibited by the repression of CPBF8

In order to further investigate whether CPBF8 is involved in trafficking of β-hexosaminidase α-subunit and lysozymes to phagosomes, we compared these activities in phagosomes isolated and purified, as previously described [12], from CPBF8gs and control strains (Figure 4E). We observed that β-hexosaminidase α-subunit and lysozyme activities in purified phagosomes decreased by 90 and 96%, respectively, in CPBF8gs, compared to the control strain, while the amylase activity in phagosomes remained unchanged. Immuno blot analysis also confirmed the results of the activity assays, and indicated that lysozyme 2 is not transported to phagosomes in CPBF8gs strain (Figure 4F).

### Repression of CPBF8 inhibits digestion of ingested bacteria

**Figure 5:**
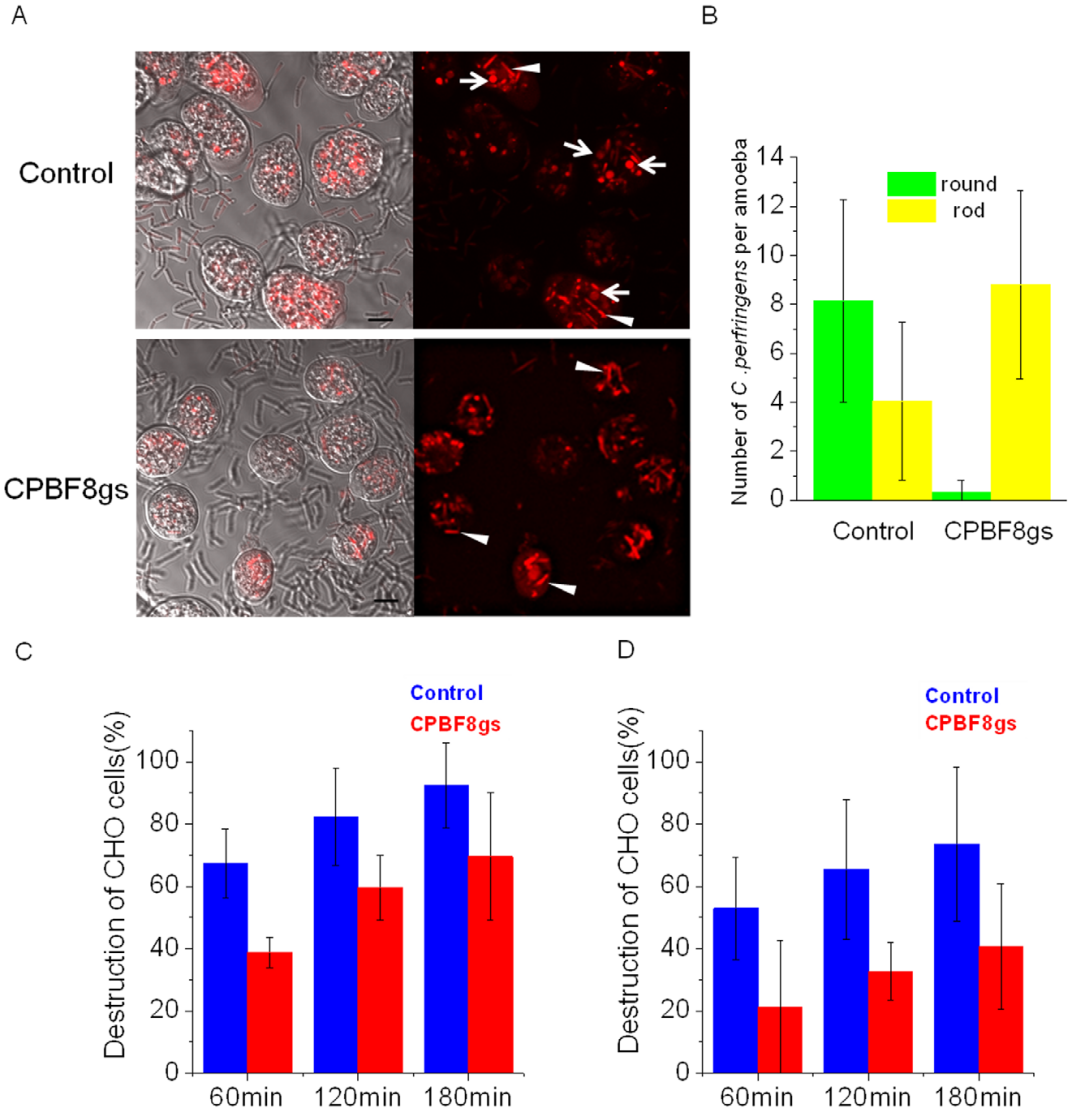
Effects of CPBF8 repression on the digestion of *C. perfringen*s and destruction of CHO monolayers. (A) Micrographic images of control and CPBF8gs strains coincubated with *C. perfringens*. Approximately 1.5×10^4^ cells of the control and CPBF8gs strains were incubated with 1.5×10^6^ *C. perfringens*, pretreated with 10 µM of SYTO-59. After 4 h co-incubation, amoebas were washed and microscopically examined. Bar, 10 µm. Arrows indicate representative round shape “deformed” or “damaged” *C. perfringens*. Arrowheads indicate representative rod-shaped “intact” *C. perfringens*. (B) Quantitative analysis of rod-shaped and round *C. perfringens* in the control and CPBF8gs strains. Data shown are the means ± standard deviations of 20 independent cells. (C, D) Kinetics of CHO cell destruction by the control and CPBF8gs strains. Approximately 5×10^4^ cells of the control and CPBF8gs strains, untreated (C) or pretreated with 200 µM of E-64 for 2 h and washed with PBS (D), were added to a monolayer of confluent CHO cells in 24 wells and incubated at 35°C for the indicated times. Data shown are the means ± standard deviations of four independent experiments. Monolayer destruction is expressed as the percentage of destroyed CHO cells. Data shown are the means ± standard deviations of four independent experiments.

To understand biological significance of CPBF8, we examined phagocytosis and degradation of a representative Gram-positive bacillus *Clostridium perfringens* in CPBF8gs strain. We microscopically monitored a course of degradation of ingested *C. perfringens* (Figure 5A). Intact and rod-shaped *C. perfringens* becomes rounded in phagosomes when it is permeabilized and degraded. After 4 h co-incubation of SYTO-59-prestained bacteria with the amoebae, both the rod-shaped and rounded bacteria were counted (Figure 5B). While the total number of bacteria ingested were comparable in the control and CPBF8gs strains (12.2±3.8 and 9.1±3.8 per amoeba, respectively), the number of rounded bacteria (0.3±0.4 per amoeba) dramatically decreased in CPBF8gs compared to the control (8.1±4.1 per amoeba), whereas that of rod-shaped bacteria increased by two fold (8.8±3.9 and 4.0±3.2, respectively). These results clearly indicate that degradation of *C. perfringens* was inhibited by the repression of CPBF8.

### Repression of CPBF8 decreases the cytopathic activity

We investigated whether CPBF8 is involved in the cytopathic effects on monolayers of cultured mammalian cells. The monolayers of Chinese hamster ovary (CHO) cells were incubated with the control and CPBF8gs strains for 1–3 h, and destruction of CHO cells was measured. The cytopathic activity caused by CPBF8gs strain was lower by 23–29% at all time points compared to control strain (Figure 5C). The observed cytopathic effect was partially blocked by 200 µM of the cysteine protease inhibitor E-64 [44], [45]. The cytopathic effect by the control strain was reduced by 20–22%, whereas that by CPBF8gs was decreased by 41–45% (Figure 5D). These results support the hypothesis that the decrease in the cytopathic activity in CPBF8gs was due to the decrease in β-hexosaminidase α-subunit and lysozymes.

To confirm this hypothesis, we also created the strains where β-hexosaminidase α-subunit or lysozyme 1 genes was repressed (HexAgs and Lys1gs strains). These silenced strains showed reduced cytotoxity to CHO cells compared to the control mock transformant by 9–18% reduction, as measured at 60 mins of co-incubation (Supplemental information Figure S2). These data indicate that secreted (and maybe also intracellular) lysozymes are involved in CHO cytolysis, and that intracellular β-hexosaminidase α-subunit is also involved in pathogenesis against mammalian cells, though its mechanism remains undetermined. We also attempted to directly test cytotoxic activity of recombinant hexosaminidase and lysozymes produced by in vitro translation (up to 10 µg/ml final), but failed to demonstrate it.

### The serine-rich region in CPBF8 is responsible for the binding with its cargo, but not its localization

**Figure 6:**
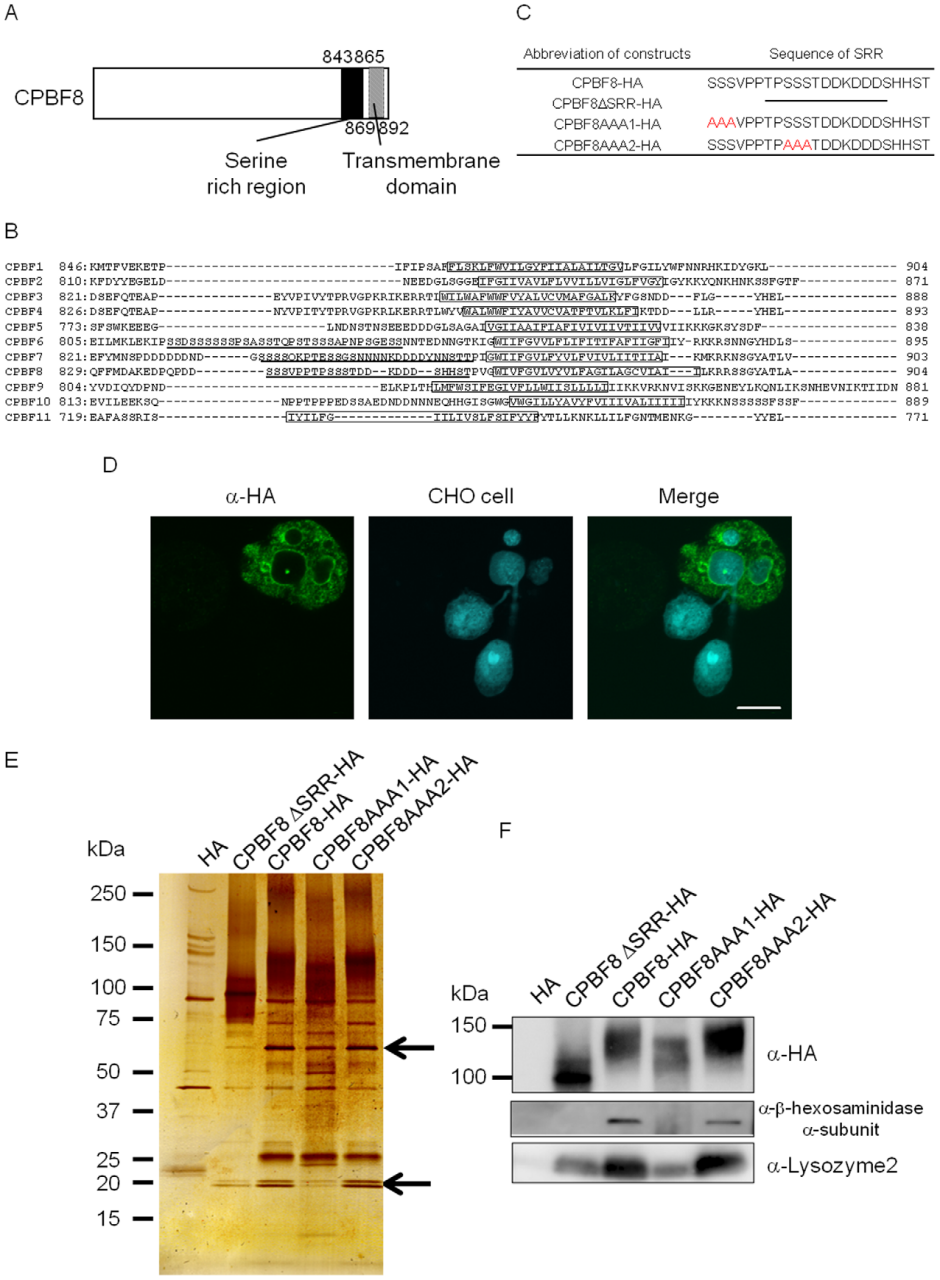
The serine-rich region of CPBF8 is involved in the binding with β-hexosaminidase α-subunit and lysozymes. (A) Schematic diagram of the serine-rich region and transmembrane domain in CPBF8. Numbers indicate amino acid positions from the amino terminus. The filled or hatched box depicts the serine rich region or the transmembrane domain, respectively. (B) Comparison of the carboxyl-terminal region of CPBF proteins. Boxes indicate the putative transmembrane domain. The serine-rich region is underlined. (C) The amino acid sequences of the wild-type and mutated serine-rich regions (SRR). Note that the entire SRR was deleted in CPBF8ΔSRR-HA. The first or second stretch of three serine residues within SRR were substituted with alanines in CPBF8AAA1-HA and CPBF8AAA2-HA, respectively. (D) Localization of CPBF8ΔSRR-HA to phagosomes. Amoebae were incubated with Cell Tracker Blue-stained CHO cells (*blue*) for 60 min, fixed, and reacted with anti-HA antibody (*green*). Bar, 10 µm. (E–F) Isolation and identification of binding proteins of CPBF8-HA, CPBF8ΔSRR-HA, CPBF8AAA1-HA, and CPBF8AAA2-HA. Lysates of CPBF8-HA, CPBF8ΔSRR-HA, CPBF8AAA1-HA, and CPBF8AAA2-HA transformants were mixed with anti-HA-antibody-conjugated agarose, washed, and eluted with HA peptide. Immunoprecipitated samples were separated on SDS-PAGE and silver stained (The upper and lower arrow indicated that β-hexosaminidase α-subunit and lysozymes, respectively. (E), or blotted and reacted with anti-HA, β-hexosaminidase α-subunit and lysozyme2 antibody (F).

CPBF family proteins show common structural organization: the signal peptide at the amino terminus, the transmembrane domain close to the carboxyl-terminal end, and the YxxL motif in the cytosolic tail located at the carboxyl terminus (Nakada-Tsukui K, et al., unpublished data). Besides, among 11 members, only 3 members, CPBF6, CPBF7, and CPBF8, have a stretch of serine-rich hydrophilic region prior to the transmembrane domain (Figure 6A and B). In order to investigate whether this region is involved in the binding of CPBF8 to the cargos and whether the region is involved in the phagosomal transport, we created a transformant that expressed HA-tagged CPBF8 lacking the 23-a.a.-long serine-rich region (CPBF8ΔSRR-HA). We immunoprecipitated CPBF8-HA and CPBF8ΔSRR-HA using anti-HA antibody from lysates of the corresponding strains. Both the silver-stained SDS-PAGE gel and immunoblot analysis with HA antibody showed that the size of CPBF8ΔSRR-HA (∼100 kDa) detected was ∼50 kDa smaller than that of CPBF8-HA (∼150 kDa), which was larger than predicted (Figure 6 E and F, see below *“The nature of post-translational modifications of CPBF8”*). The amount of the 75- and 25-kDa proteins, which correspond to β-hexosaminidase α-subunit and lysozymes, respectively, detected in the immunoprecipitated samples from the lysates of CPBF8ΔSRR-HA significantly decreased, compared to that from CPBF8-HA strain (Figure 6E and F). The identity of the precipitated proteins was confirmed by the immunoblots using anti-β-hexosaminidase α-subunit antibody and lysozyme 2 antibody (Figure 6F).

To further map the region and amino acids responsible for the cargo binding, we constructed the variant forms of CPBF8 in which one of two stretches of three serines in the SRR were replaced by alanines (CPBF8AAA1-HA and CPBF8AAA2-HA, respectively) (Figure 6C). CPBF8AAA1-HA showed reduced ability to bind β-hexosaminidase α-subunit and lysozyme 2, compared to CPBF8-HA and CPBF8AAA2-HA (Figure 6E and F). These data indicate that the region containing the first stretch of three serine residues is essential for the binding with the cargos. Furthermore, silver staining and immunoblots with anti-HA antibody of the lysate from CPBF8AAA1-HA showed a ∼20 kDa reduction in the apparent molecular size of CPBF8AAA1-HA, compared to CPBF8-HA. These data were also consistent with a premise that this portion is directly post-translationally modified or indirectly involved in post-translational modifications (see below). We also attempted to show direct evidence that β-hexosaminidase α-subunit and lysozymes bind to SRR by constructing a truncated form of CPBF8, in which the signal peptide, SRR, the transmembrane domain, and the cytosolic region of CPBF8 were included (designated as SRR-HA). However, neither β-hexosaminidase α-subunit nor lysozymes was detected by immunoprecipitation of SRR-HA (data not shown). This indicates that the SRR per se may not be sufficient for post-translational modifications required for cargo binding. Immunofluorescence assay showed that the localization of CPBF8ΔSRR-HA, CPBF8AAA1-HA, CPBF8 AAA2-HA, and SRR-HA such as phagosome recruitment (Figure 6D and Supplemental information Figure S3A–C) was indistinguishable from that of CPBF8-HA. Therefore, SRR does not appear to be essential to phagosome targeting.

### The nature of post-translational modifications of CPBF8

**Figure 7:**
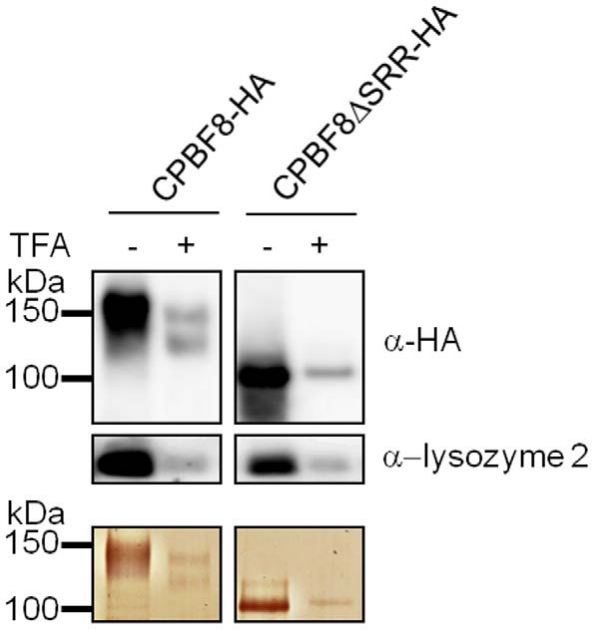
Post-translational modification of CPBF8. CPBF8-HA and CPBF8ΔSRR-HA was immunoprecipitated with anti-HA antibody from the lysates of CPBF8-HA and CPBF8ΔSRR-HA transformants, treated (+) or untreated (−) with TFA for 10 min. The samples were separated on SDS-PAGE and silver-stained (bottom panel), or blotted and reacted with anti-HA (top panel) or anti-lysozyme 2 antibody (middle panel). The apparent molecular weight (kDa) of standards are indicated on the left.

The apparent molecular mass of CPBF8ΔSRR-HA detected with silver staining and immunoblots with anti-HA antibody was ∼50 kDa smaller than that of CPBF8-HA (Figure 6, E and F, Figure 7). The reduction of the apparent size was larger than the predicted decrease based on the deletion of the amino acids (23 a.a. corresponding to 2.4 kDa for CPBF8ΔSRR-HA). To better understand the nature of the post-translational modification of CPBF8, we treated the transformant with 10 µg/ml tunicamycin, which is an inhibitor of asparagine-linked glycan modification, for 24 h (40). However, tunicamycin treatment did not affect the apparent mobility of immunoprecipitated CPBF8-HA on SDS-PAGE (data not shown), despite the fact that a potential N-linked glycosylation site is present in CPBF8 (Asn383). It was previously shown that the major GPI-anchored surface antigen of *E. histolytica* trophozoites contains O-phosphodiester-linked sugars [46]. We examined if the post-translational modification of CPBF8 contains O-phosphodiester-linked sugars by the treatment of immunoprecipitated CPBF8 with trifluoroacetic acid (TFA). SDS-PAGE and immunoblot analyses showed that TFM treatment of immunoprecipitated CPBF8 reduced the apparent molecular size of CPBF8 to ∼120 kDa (Figure 7), which was similar to the size of CPBF8AAA1-HA (Figure 6, E and F), while that of CPBF8ΔSRR-HA remained unchanged by TFA treatment. Altogether, CPBF8 appears to possess O-phosphodiester linked carbohydrates via the first stretch of serines within SRR, and this region seems to be responsible for the binding with β-hexosaminidase α-subunit and lysozymes. It should also be noted that the apparent size of TFA-treated CPBF8-HA and CPBF8AAA1-HA was significantly (∼20 kDa) larger than that of CPBF8ΔSRR-HA. The difference between CPBF8AAA1-HA and CPBF8ΔSRR-HA was apparently larger than the predicted size of SRR (2.4 kDa), suggesting that other post-translational modification(s) may be present in other region(s) of SRR.

## Discussion

### Discovery of a novel transport receptor of β-hexosaminidase α-subunit and lysozymes

CPBF8 was first identified in phagosomes by our previous proteome study of the purified phagosomes [12]. We have recently rediscovered CPBF8 as a homolog of CPBF1. CPBF1 was isolated as a potential receptor/carrier of the major virulence factor of *E. histolytica*, CP5, by virtue of its binding activity to CP5 (Nakada-Tsukui K, et al., unpublished data). CPBF8 represents a novel hydrolase receptor for the following reasons. First, CPBF8 is the first receptor that binds to and transport β-hexosaminidase α-subunit and lysozymes to lysosomes/phagosomes, in the manner that is distinct from mannose-6-phosphate receptor- and sortilin-dependent pathway. Second, there is no CPBF8 homolog in other organisms, and showed no sequence similarity to mannose-6-phosphate receptors or sortilin at the primary sequence level. Mannose-6-phosphate receptor and sortilin 1 are the membrane receptor of cathepsin D/β-hexosaminidase [47] and prosaponin/sphingolipid activator protein [48], respectively. Third, CPBF8 is post-translationally modified at its unique serine-rich region, and the modification is essential for the cargo binding.

### Cellular localization of CPBF8

The two representative CPBF members, CPBF1 and CPBF8, are localized to distinct compartments in steady state. Immunofluorescence assay using two markers, LysoTracker and PNT, clearly showed distinct distribution of CPBF1 and CPBF8. CPBF8 was well colocalized with LysoTracker and PNT (Figure 1), whereas CPBF1 was seldom localized to lysosomes or colocalized with PNT (Nakada-Tsukui K, et al., unpublished data). The different localization of CPBF8 and CPBF1 may be attributable to the motif sequences at the carboxyl terminus. As mentioned above, the YxxL motif is located at the very end of the carboxyl terminus CPBF1, while CPBF8 ends with a stretch of YxxLA, suggesting a possibility that different accessory molecule(s) bind to CPBF 1 and CPBF8.

When CPBF1 and CPBF8 were recruited to phagosomes, the subdomain of the phagosomal membrane they first come in contact with, seems to be indistinguishable. CPBF8 (Figure 1A) and CPBF1 (Nakada-Tsukui K, et al., unpublished data) were recruited to the basolateral portion of the “phagocytic mouth”, similar to the domain where phosphatidylinositol-3-phosphate is localized [35]. The fact that CPBF8 and *Eh*PNT colocalize in steady state and are simultaneously transported to phagosomes upon phagocytosis, suggests that similar trafficking pathway and mechanism may be used despite the apparent difference in the predicted strength of the membrane association (one versus 11–13 transmembrane regions, respectively). Further experiments are necessary to identify a key factor that determines cellular localization.

### Cargo specificity and physiological function of CPBF8

Affinity immunoprecipitation of CPBF8 led us to identify β-hexosaminidase α-subunit and lysozymes as the major cargos of CPBF8. The cargo specificity of CPBF8 was further supported by the dramatic reduction of their enzymatic activities in both the whole cell and phagosomes, by repression of *CPBF8* gene. The observation is consistent with the previous finding on the mouse fibroblast, in which knockout of a specific receptor caused decrease in the intracellular activities of β-hexosaminidase, β-galactosidase, and β-glucuronidase [47].

Although deletion or repression of hydrolase receptors often causes missecretion of cargos (e.g., [47]), repression of *CPBF8* gene did not result in missecretion of β-hexosaminidase α-subunit and lysozymes. This is also in good contrast to CPBF1, gene silencing of which caused missecretion of CP5 (Nakada-Tsukui K, et al., unpublished data). The outcome of deletion or repression of a hydrolase receptor largely varies. These data suggest that the trafficking, processing, activation, secretion, and degradation of cysteine proteases and β-hexosaminidase α-subunit/lysozymes largely differ despite they use the transport receptors that belong to the same protein family. It is likely that non- or mis-targeted β-hexosaminidaseα-subunit and lysozymes remain inactive or are swiftly degraded by proteasomes.

Lysozymes are the well-established anti-bacterial protein, which degrades the cell wall of Gram-positive bacteria. Thus, the reduction of destruction of *C. perfringens* caused by CPBF8gs can be directly attributable to the loss of lysozymes in phagosomes. It has also been reported that β-hexosaminidase in *Drosophila melanogaster* [49] and in murine macrophages is important to repress and control the growth of *Mycobacterium marinum* [49]. Therefore, the defect in the transport of β-hexosaminidase α-subunit to phagosomes may also be responsible for the decrease in degradation of *C. perfringens* in CPBF8gs strain.

### Phagosomal transport of hydrolases via CPBF8 contributes to the cytopathic activity on mammalian cells

We have shown that the cytopathic effects of trophozoites were decreased by repression of *CPBF8*, and the reduction of the cytopathy was not due to cysteine proteases, as the decrease in the cytopathy caused by *CPBF8* gene silencing was not cancelled by the cysteine protease inhibitor. These results support the premise that the enzymatic activity that decreased in CPBF8gs strain, i.e., β-hexosaminidase α-subunit and/or lysozymes, is responsible for the cytopathic effect remaining after E-64 treatment. The present study is the first to show the causal link of β-hexosaminidase α-subunit and lysozyme with virulence in eukaryotic pathogens. In mammals, β-hexosaminidase is known to hydrolyze GM2 (sphingomyelin) [6]. Recently, it has also been shown that β-hexosaminidase is involved in fertilization in hamster [50]. In addition, β-hexosaminidase from the Asian corn borer *Ostrinia furnacalis* was shown to degrade chitin [51]. Phylogenetic analysis indicated that both α and β-subunit of β-hexosaminidase from *E. histolytica* belong to the same clade as insect counterparts [52]. This clade contains two different functional β-hexosaminisases from insects. One is involved in the alteration of the structure of N-glycans generated in the cell, while the other plays in the chitin degradation processes. Although it has not been demonstrated how β-hexosaminidase is involved in the cytopathy of *E. histolytica*, it is conceivable that *E. histolytica* β-hexosaminidase degrades glycoconjugates of the extracellular matrix components to pass basement membranes, as previously suggested [53]. It was reported that lysozyme gene was poorly expressed in *E. histolytica* Rahman strain, which apparently lost virulence, and non-virulent *E. dispar*, compared to *E. histolytica* HM-1:IMSS strain [54], [55]. Furthermore, lysozyme was also poorly expressed in *E. histolytica* trophozoites that were treated with 5-azacytidine (5-AzaC), a potent inhibitor of DNA methyltransferase, and showed reduced virulence [56]. Altogether, lysozymes are involved in *in vitro* cytotoxicity and *in vivo* virulence in *E. histolytica*.

The fact that CP was responsible for only 15–25% of the cytopathic effect on CHO cells in control transformant apparently disagreed to our previous finding [29], where 70–75% of the cytopathic effect in the HM-1 reference strain was attributable to CP. This is likely explained by the fact that the parental strain (G3) of the transformants for gene silencing has uncharacterized defects as well as lack of amoebapores.

### Structural determinants of cargo binding in CPBF8

As mentioned above, the most striking difference between non-lysosomal/phagosomal CPBF1 and lysosomal/phagosomal CPBF8, at the primary sequence level is the serine-rich domain in the luminal region, found exclusively in CPBF6, 7, and 8. While either the deletion of this region or point mutations of the serine stretch of the serine-rich region did not affect trafficking to phagosomes, the binding of CPBF8 to β-hexosaminidase α-subunit and lysozymes was significantly reduced. Although we cannot exclude the possibility that truncated CPBF8 was partially mis-folded and thus unable to bind to its cargos, characterization of the post-translational modifications of CPBF8 via the serine-rich region by chemical removal of its potential *O*-phosphodiester-linked glycans strongly indicates that the O- phosphodiester-linked glycan within this region appears to be involved in cargo binding.

In summary, we have discovered and characterized the novel membrane-associated receptor for β-hexosaminidase α-subunit and lysozymes, CPBF8, from *E. histolytica*. We have demonstrated that CPBF8 plays an important role in the degradation of ingested bacteria in phagosomes and the cysteine protease-independent cytopathy on mammalian cells.

## Materials and Methods

### Microorganisms and cultivation

Trophozoites of *E. histolytica* strain HM-1:IMSS Cl-6 and G3 [41] were cultured axenically at 35°C in 13×100 mm screw-capped Pyrex glass tubes or plastic culture flasks in BI-S-33 medium as previously described [57], [58]. CHO cells were grown in F-12 medium (Invitrogen, Grand Island, NY) supplemented with 10% fetal bovine serum on a 10-cm-diameter tissue culture dish (IWAKI, Tokyo, Japan) under 5% CO2 at 37°C. *Clostridium perfringens* was kindly given by Fumiya Kawahara, Nippon Institute for Biological Science Japan.

### Plasmid construction and production of *E. histolytica* transformants

The protein coding region of *CPBF8* gene was amplified by PCR from cDNA using sense and antisense oligonucleotides: 5′-GCGAGATCTATGTTGGCACTCTTCGCCATC-3′ and 5′-GCGAGATCTAGCTAAAGTAGCATATCCAGA-3′ (BglII restriction sites are underlined). The amplified PCR product was digested with BglII and ligated into BglII-digested pEhExHA [28], to produce pEhEx-CPBF8-HA. The plasmid to produce the mutant form of CPBF8 that lacks 23a.a.-long serine-rich region (pEhEx-CPBF8ΔSRR-HA) was constructed as follows. A DNA sequence was amplified by PCR from pEhEx-CPBF8-HA using sense and antisense oligonucleotides: 5′-CCAGTTGGATGG- ATTGTATTTGGTGTTCTT-3′ and 5′-GTCATCTGGTTGTGGATCTTC TTTAGCATC-3′. The PCR product was treated with BKL kit (Takara, Shiga, Japan), and self-ligated to produce pEhEx-CPBF8ΔSRR-HA. The resulting plasmid encodes a mutant CPBF8 protein lacking 843–865 a.a.-region (“SSSVPPTPSSSTDDKDDDSHHST”). Two variant forms of CPBF8 containing point mutations, S843A, S844A, and S845A, or S851A, S852A, and S853A, respectively (designated as CPBF8AAA1-HA and CPBF8AAA2-HA), are constructed as follows. DNA fragments corresponding to the portion of the protein coding region encompassing from the amino-terminal region to the mutated amino acids of CPBF8AAA1-HA and CPBF8AAA2-HA were amplified by PCR from pEhEx-CPBF8-HA, using sense and antisense oligonucleotides: 5′-ACAAACACATTAACAATGTTGGCACTCTTCGCC -3′ and 5′-TGGTACTGCAGCAGCGTCATCTGGTTGTGG -3′ (for CPBF8AAA1-HA) and 5′-ACAAACACATTAACAATGTTGGCACTCTTCGCC -3′ and 5′-ATCAGTTGCAGCAGCTGGTGTTGGTGGTAC -3′ (for CPBF8AAA2-HA), where the nucleotides corresponding to the mutated amino acids (serine to alanine) are double-underlined). DNA fragments corresponding the portion of the protein coding region encompassing the mutated amino acids of to the carboxyl-terminal region of CPBF8AAA1-HA and CPBF8AAA2-HA were amplified by PCR from pEhEx-CPBF8-HA using sense and antisense oligonucleotides: 5′- GCTGCTGCAGTACCACCAACACCATCTTC-3′ and 5′- ATCATATGGATACATAGCTAAAGTAGCATATCC -3′ (for CPBF8AAA1-HA), and 5′- GCTGCTGCAACTGATGATAAAGATGATG -3′ and 5′- ATCATATGGATACATAGCTAAAGTAGCATATCC-3′ (for CPBF8AAA2-HA), where the nucleotides corresponding to the mutated amino acids (serine to alanine) are double-underlined. The two amplified fragments were mixed and ligated to BglII-digested pEhExHA using GENEART Seamless ligation kit (Invitrogen, San Diego, CA, USA), to produce pEhEx-CPBF8AAA1-HA and pEhEx-CPBF8AAA2-HA. SRR-HA, which consisted of the signal peptide, SRR, the transmembrane domain, and the cytosolic region of CPBF8, was constructed as follow. A DNA fragment corresponding to the signal peptide and additional four amino acids was amplified by PCR using sense and antisense oligonucleotides: 5′- ACAAACACATTAACAATGTTGGCACTCTTCGCC -3′ and 5′- AGAACATGTGTACTGTCCATACGCAAC -3′. A DNA fragment corresponding to SRR, the transmembrane domain, and cytosolic region was amplified by PCR using sense and antisense oligonucleotides: 5′- CAGTACACATGTTCTGATGACTCTTCTTCAGTACC -3′ and 5′- ATCATATGGATACATAGCTAAAGTAGCATATCC-3′. The two amplified fragments were mixed and ligated to BglII-digested pEhExHA using GENEART Seamless ligation kit to produce pEhEx-SRR-HA. The transformants that expressed CPBF8-HA, CPBF8ΔSRR-HA, CPBF8AAA1-HA, CPBF8AAA2-HA, or SRR-HA were established by transfection of the wild-type HM1:IMSS Cl6 strain by liposome-mediated transfection as previously described [59].

Repression of gene expression was accomplished by gene silencing, which has recently demonstrated to be mediated by nuclear localized antisense small RNAs with 5′-polyphosphate termini [42]. Gene silencing has been seen only in G3 strain, in which amoebapore genes are repressed. Thus, whatever phenotypic changes are observed by gene silencing of additional gene of interest, need to be compared against the control G3 strain transformed by the mock gene silencing plasmid (pSAP2-Gunma). Furthermore, it should be evaluated, if possible, whether the phenotypic changes are not caused by synergistic effects with amoebapore silencing. For gene silencing of *CPBF8, β-hexosaminidase α-subunit*, and *lysozyme 1* genes, the 420-bp-long 5′-end of the protein coding region was amplified by PCR from cDNA using sense and antisense oligonucleotides: 5′-CGCAGGCCTATGTTGGCACTCTTCGCCATC-3′ and 5′-GCAGAGCTCATTTTCTTCAACTAACTTAAC-3′ (*CPBF8*); 5′-CGCAGGCCTATGCCATATCCAAGCTCAG-3′ and 5′-CGCGAGCTCGTTTGATGAAATTCTAATT-3′ (*β-hexosaminidase α-subunit*); 5′-CGCAGGCCTATGTTCGCTCTCTTTTTGTG-3′ and 5′-CGCGAGCTCACCATGGACAATACCAATAGC-3′ (*lysozyme 1*) (StuI and SacI restriction sites are underlined). The PCR-amplified DNA fragment was digested with StuI and SacI, and ligated into StuI- and SacI-digested pSAP2-gunma [60], to produce pSAP2-CPBF8, pSAP2-HexA, and pSAP2-Lys1. The gene-silenced strains were established by the transfection of G3 strain with the corresponding plasmids as described above.

### Antibodies

CPBF8 and lysozyme 2 antibodies were raised against recombinant histidine-tagged partial CPBF8 (a.a. 14–292) and full-length lysozyme 2, respectively. The method of production and purification of recombinant proteins were described before [40]. The expression plasmids for histidine-tagged CPBF8 (a.a.14–292) and lysozyme 2 were introduced into BL21(DE3) competent cells (Invitrogen). Expression of the recombinant protein was induced with 0.1 mM isopropyl-β-thiogalactoside at 37°C for 3 h. The histidine-tagged fusion protein was purified under denaturing condition using Ni-NTA agarose (QIAGEN, Hiden, Germany), according to the manufacturer's protocol. β-hexosaminidase α-subunit antibody was raised against mixture of the peptides LQQQTGLQDFKVSL (a.a. 77–90) and GWSKSKEYSDIQKF (a.a. 348–361).

Anti-HA 11MO mouse monoclonal antibody was purchased from Berkeley Antibody (Berkeley, CA). Alexa Fluor anti-mouse and antirabbit IgG and horseradish peroxidase (HRP)-conjugated goat anti-mouse were purchased from Invitrogen.

### Immunofluorescene assay

For the staining of lysosomes, amoebae were incubated in the BI-S-33 medium containing LysoTracker Red DND-99 (Invitrogen) (1∶500) at 35°C for 12 h. To visualize phagosomes, CHO cells were pre-stained with 10 βM of CellTracker Blue (Invitrogen) in F-12 medium supplemented with 10% fetal bovine serum at 37°C for 3 h. Labeled CHO cells were washed with phosphate-buffered saline (PBS), and added to 8-mm wells containing *E. histolytica* trophozoites on a slide glass (8 well 8 mm standard slide glass, Thermo Scientific, Rockford, IL) and further incubated at 35°C for 10–60 minutes. After the incubation, cells were fixed with 3.7% paraformaldehyde for 10 min, and permeabilized with 0.2% saponin/PBS for 10 min at ambient temperature. The cells were then reacted with anti-HA 11MO mouse monoclonal antibody (diluted at 1∶1000) and Alexa Fluor-488 antimouse secondary antibody (1∶1000). The samples were examined on a Carl-Zeiss LSM510 conforcal laser-scanning microscope (Thornwood, NY). Images were further analyzed using LSM510 software. We defined CPBF8-HA- and LysoTracker-positive and negative vacuoles/vesicles as follows: 1) we measured and averaged the signal intensity (per pixel) of the whole intracellular area of a cell, and also five randomly-chosen areas outside the cell to obtain a background fluorescence level; 2) for individual vacuoles/vesicles that had continuous fluorescent signal lining the membrane, two straight lines were drawn, which make a right (90-degree) angle, and a projected fluorescence histogram was obtained for each line; 3) if the peak intensity of the point on the membrane of the vacuole/vesicle was >2 fold of the background fluorescence level, the vesicle/vacuole was defined as “signal positive”. The LysoTracker-positive vacuoles/vesicle was defined similarly, except that 1) the fluorescence of not a point on the membrane, but a whole intravesicular/vacuolar area was measured and averaged; and 2) the threshold of the average peak intensity of the LysoTracker-positive area in the vacuole/vesicle is >5 fold of the background fluorescence level.

### Immunoprecipitation

Approximately 3×10^6^ cells of CPBF8-HA-expressing amoebae were lysed in 2 ml of lysis buffer [50 mM Tris-HCl, pH 7.5, 150 mM NaCl, 1% Triton X-100 (Tokyo Kasei, Tokyo, Japan), 0.5 mg/ml E-64 (Sigma-Aldrich, St. Louis, MO)], and suitable amount of Complete mini mix (Roche, Barsel, Switzerland), and was incubated with protein G-Sepharose beads (50 µl of a 80% slurry) (Amersham Biosciences, Uppsala, Sweden) at 4°C for 90 min, centrifuged at 800× g at 4°C for 3 min to remove proteins that bind to the protein G-Sepharose beads non-specifically. The precleaned lysate was mixed with 90 µl of anti-HA-conjugated agarose (50% slurry-, Sigma-Aldrich), and incubated at 4°C for 3.5 h. The agarose beads were collected by centrifugation at 800× g at 4°C for 3 min, and washed four times using wash buffer (50 mM Tris-HCl, pH 7.5, 150 mM NaCl, 1% Triton-X 100). The agarose beads were then incubated with 180 µl of HA peptide (20 µg/ml) at 4°C for overnight to dissociate proteins from the beads. The eluate was applied to SDS-PAGE and silver staining as previously described [61].

### Protein digestion, LC–MS, and MS/MS

Silver stained gels were excised, destained, and tryptic-digested using modified trypsin (Applied Biosystems Darmstadt, Germany). Briefly, excised gels were transferred to a siliconized tube, dehydrated in acetonitrile, rehydrated in 30 µl of 10 mM dithiothreitol in 0.1 M ammonium bicarbonate and reduced at room temperature for 30 min. The sample was then alkylated in 30 µl of 50 mM iodoacetamide in 0.1 M ammonomium bicarbonate at room temperature for 30 min. The reagent was removed and the sample was dehydrated in 100 µl acetonitrile, rehydrated in 100 µl of 0.1 M ammonium bicarbonate, and then dehydrated again in 100 µl acetonitrile and completely dried by vacuum centrifugation. Samples were then rehydrated in 20 ng/ml trypsin in 50 mM ammonium bicarbonate on ice for 10 min. Any excess trypsin solution was removed and 20 µl of 50 mM bicarbonate added. The samples were digested overnight at 37°C and resultant peptides were extracted in two 30 µl aliquots of 50% acetonitrile/5% formic acid. The tryptic peptides were eluted from the gel and then desalted by Ziptip. The resulting peptide mixture was separated by reverse phase chromatography (DiNa nano LC system; KYA Tech, Tokyo, Japan) using a 0.15 mm×50 mm ID HiQ sul C18W3 column (KYA Tech) and elution with 0.1% formic acid/2% CH3CN (solvent A) and 0.1% formic acid/80% CH3CN (solvent B) using a program 0% solvent B for 15 min, gradient at 4%/min for 2 min, gradient at 0.86%/min for 43 min, 11%/min for 5 min, 100% solvent B for 10 min with a total flow rate of 300 nl/min. The eluting peptides were ionized by electrospray ionization and analyzed by a 3200 Q TRAP LC/MS/MS System (Applied Biosystems). Peptide MS/MS spectra were acquired in an information-dependent manner using the Analyst QS software 2.0 acquisition features (Smart Exit, rolling collision energy, and dynamic exclusion).

### Database search

Peptide sequence data obtained by mass spectrometry were analyzed against the *E. histolytica* genome database at The Institute for Genomic Research (TIGR) (http://www.tigr.org/tdb/e2k1/eha1/) using the Sequest algorithm. Sequencing data were also analyzed against the non-redundant database at the National Center of Biotechnology Information (NCBI). Individual predicted protein sequences were manually analyzed by BLAST search (http://www.ncbi.nlm.nih.gov/BLAST/) against the non-redundant database at NCBI. The identification of the protein was considered significant when at least two non-overlapping peptides of a protein were detected with the probability score >95%. The identified proteins were classified using the annotations provided in the TIGR and NCBI database and results of BLAST search.

### RT-PCR

Total RNA was extracted using TRIzol reagent (Invitrogen) according to the manufacturer's instructions. The synthesis of cDNA was performed using the SuperScript III First Strand Synthesis System (Invitrogen) according to the manufacturer's instructions. The cDNA synthesis was completed on a DNA Engine Peltier Thermal Cycle (Bio-Rad Laboratories, Inc., Hercules, CA, USA) and treated with deoxyribonuclease I (Invitrogen) to exclude genomic DNA. PCR was performed with the resulting cDNA as a template and specific oligonucleotide primers. Primers used were 5′ (-CAAGTGCTTCAAGTACTCAACCATC-3′and 5′-ACCATTGTTACTTCTCTTTTTACGA-3′ (*CPBF6*); 5′-ATTTTATATGAACTCTCCAGACGAT-3′ and 5′-TAATAGTAATAATCAGCACAATAAC-3′ (*CPBF7*). 5′-AGAAGGTTTTGTCGATGTTCAATTC-3′ and 5′-TAACACATCCAGCAAGAATACCAGC-3′ (*CPBF8*). PCR reaction mixture contains 0.2 U of high-fidelity DNA polymerase (Phusion, FINNZYME, Espoo, Finland), 5×Phusion buffer, 0.16 mmol/L of each dNTPs, 0.5 µM Primers. Parameters for PCR were: an initial step of denaturation at 98°C for 30 sec, 25 cycles of amplification (at 98°C for 5 sec, 53°C for 20 sec, and 72°C for 2 min), and a final extension at 72°C for 5 min.

### Microarray analysis

Expression analysis was performed using a custom *E. histolytica* array from Affymetrix, Inc. (Santa Clara, CA, USA), as previously described [62]. Labeled cRNA for hybridization was prepared from 5 µg of total RNA according to published Affymetrix protocol. Hybridization and scanning were performed according to Affymetrix protocols.

### Preparation of cell lysates and culture supernatants

A semi-confluent culture was harvested at 48–72 h after initiation of the culture and resuspended in modified Opti-MEM (Invitrogen), Opti-MEM supplemented with 1 mg/ml ascorbic acid and 5 mg/ml cysteine. Approximately 4×10^5^ amoebae in 1 ml of the medium were seeded to wells of a 12-well plate. After the culture was incubated at 35.5°C for 2 h, the culture supernatant was centrifuged at 400× g for 5 min at 4°C to remove debris. The plates were chilled on ice for 5 min and detached trophozoites were collected.

### Phagosome purification

Approximately 3–5×10^6^ trophozoites (per flask) were cultured in 25-cm^2^ flasks for 48 h, and washed gently with warm modified Opti-MEM. Approximately 10^7^ carboxylate-modified latex beads (Polyscience, Warrington, PA) were added to the flasks, and the flasks were centrifuged at 190× *g* for 5 min to bring the beads into contact with the trophozoites. After centrifugation, the flasks were placed on ice for 10 min. The trophozoites were washed three times with cold PBS containing 20% sucrose, followed by centrifugation at 190× *g* for 5 min to remove uningested beads. The cells containing latex beads were then resuspended in warm BI-S-33 medium, further incubated at 37°C, and harvested after 120 min. Bead-containing phagosomes were purified as previously described [12] with some modifications. Briefly, after harvesting, the amoebae that contained latex beads were resuspended in cold homogenization buffer (250 mM sucrose, pH 7.4, 3 mM imidazole, 10 mM cysteine protease inhibitor E-64, CompleteMini protease inhibitor cocktail) and homogenized with a Dounce homogenizer on ice. Phagosomes containing latex beads were then separated by flotation on a sucrose step gradient as described [12]. All sucrose solutions were made in 3 mM imidazole, pH 7.4 containing 10 mM cysteine protease inhibitor E-64. Sucrose was added to the homogenized lysate to 40%. 2 ml of the lysate containing 40% sucrose, 2 ml each of 35, 25 and 10% sucrose solutions were carefully overlaid in a 10 ml ultracentrifuge tube. The sample was centrifuged in a swinging bucket rotor MLS-50 (Beckman, Brea, CA) at 131,000× *g* at 4°C for 1 h. The phagosome fraction was collected from the interface of the 10 and 25% sucrose solutions. The collected fraction (1 ml) was mixed with 3 ml of 50% sucrose, and transferred to a new tube. To the sample, 4 ml of 25% and 2 ml of 10% sucrose solutions were overlaid, and the sample was centrifuged at 131,000× *g* at 4°C for 1 h. The separated phagosome sample, collected from the interface of the 10 and 25% sucrose solutions, was finally mixed with the same volume of 3 mM imidazole solution and centrifuged at 13,000× *g* at 4°C for 5 min. The pellet were suspended with 7% sucrose solution and stocked in −80°C.

### Enzymatic assay

β-hexosaminidase assay was performed as previously described [43] with some modifications. Briefly, the reaction mixture consisted of 25 mM citrate buffer, pH 4.0, 10 mM 4-methylumbelliferyl-6-sulfo-2-acetamido-2-deoxy-β-D-glucopyranoside (MUGS)(Merck, Darmstadt, Germany) or 4-methylumbelliferyl -2-acetamido-2-deoxy-β-D-glucopyranoside (MUG)(Sigma) as substrates, and amoeba cell lysates, culture supernatant or phagosome fraction. The reaction was initiated by addition of the substrates and stopped by addition of 0.2 M glycine/0.2 M sodium carbonate, pH 10.5). The fluorescence of the released 4-methylumbelliferone was measured at excitation and emission wavelengths of 360 and 440 nm, respectively. The lysozyme assay was performed by EnzChek Lysozyme Assay Kit (Invitrogen). Briefly, the reaction mixture contained amoeba lysates, culture supernatant or phagosomal fraction and 200 µg/ml of Bodipy-conjugated *Micrococcus lysodeikticus* cell wall, and the fluorescence was measured at excitation and emission wavelengths of 485 and 530 nm, respectively. The amylase assay was performed by EnzChek amylase Assay Kit (Invitrogen). Briefly, the reaction mixture contained amoeba lysates, culture supernatant or phagosomal fraction, and 200 µg/ml of substrate solution, and the fluorescence was measured at excitation and emission wavelengths of 485 and 530 nm.

### Digestion assay of *C. perfringens*

Approximately 1.5×10^4^ trophozoites of control or CPBF8gs strain and 1.5×10^6^ *C. perfringens* were incubated in 150 µl of BIS medium with 10 µM SYTO-59 (Invitrogen) on glass bottom culture dish(Mattek, MA, USA) under anaerobic condition for 4 h. After incubation, the cell was washed with BIS and observed by microscopy under anaerobic condition on a Carl-Zeiss LSM510 conforcal laser-scanning microscope. The numbers of rod and round shape bacteria are counted. Images were further analyzed using LSM510 software.

### *In vitro* translation

The soluble region of lysozyme 1 and 2 were expressed by TN SP6 High-Yield Wheat Germ Protein Expression System (Promega, WI, USA). The HA-tagged lysozyme 1 and 2 were amplified by PCR from cDNA using sense and antisense oligonucleotides: 5′- GGGGCGATCGCATGTATCCATATGATGTTCCAGATTATGCTAAATTAGGTATTGATGTCTCTC -3′ and 5′- GGGGTTTAAACTTATGGTTTGTAGTTATAATC -3′ (for HA-lysozyme 1) and 5′- GGGGCGATCGCATGTATCCATATGATGTTCCAGATTATGCTGTAGATGTATCTCAACC -3′ and 5′- GGGGTTTAAACTTAAAAATTAAATAAAAAGAAATGAG -3′ (for HA-lysozyme 2), where PmeI and SgfI restriction sites are underlined and the sequence corresponding to the HA-tag are double underlined, respectively. The PCR-amplified DNA fragments were digested with PmeI and SgfI, and ligated into PmeI- and SgfI-digested pF3A WG (BYDV) Flexi vector, to produce pF3A-HA-lysozyme 1 and 2. Expression of HA-lysozyme 1 and 2 was performed according to the manufacturer's protocol. The reactions were done at 25°C for 2 h. The purification of HA-lysozyme 1 and 2 was performed as described above for immunoprecipitation.

### Cytopathic activity

CHO monolayer destruction was measured as described previously with minor modifications [63]. Briefly, CHO cells were grown with confluent in 24 well plate for over-night.at 37°C and 5% CO_2_.The medium was removed and the plates were washed with modified Opti-MEM medium. Approximately 5×10^4^ trophozoites of control or CPBF8gs strains were resuspended in 0.5 ml of modified Opti-MEM medium and added to each well. The plates were incubated under anaerobic conditions at 35.5°C for up to 3 h. The plates were placed on ice for 10 min to detach trophozoites. The number of CHO cells remaining in the wells was measured by WST-1 reagent (Roche) as described previously [64]. The cytopathic activity of recombinant HA-lysozyme 1 and 2 toward mammalian cells was evaluated by incubating confluent CHO cells with the mixture of purified recombinant proteins and 10% FBS/F-12 (1∶99) on a 24 well plate. The plates were incubated at 35.5°C for 24 h. The number of CHO cells remaining in the wells was estimated by WST-1 reagent.

### Accession numbers

CPBF1, EHI_164800, XP_655218; CPBF2, EHI_087660, XP_653276; CPBF3, EHI_161650, XP_649180; CPBF4, EHI_012340, XP_655897; CPBF5, EHI_137940, XP_654065; CPBF6, EHI_178470, XP_653036; CPBF7, EHI_040440, XP_649361; CPBF8, EHI_059830, XP_652899; CPBF9, EHI_021220, XP_655360; CPBF10, EHI_191730, XP_649015; CPBF11, EHI_118120, XP_656044; β-hexosaminidase α-subunit, EHI_148130, XP_657529; Lysozyme 1, EHI_199110, XP_653294; Lysozyme 2, EHI_096570, XP_656933

## Supporting Information

Figure S1 Growth kinetics of control and CPBF8gs strains. Approximately five thousands amoebae were inoculated to 6-ml culture medium. The amoebae of each strain were counted every 24 hours. Data shown are the means ± standard deviations of five biological replicates.
(TIF)

Figure S2 Confirmation of gene silencing in β-hexosaminidase α-subunit (HexAgs) and lysozyme 1 (Lys1gs) gene-silenced strains and their ability for the destruction of CHO monolayers. (A) Reverse transcriptase-PCR analysis. A 420-bp long partial *HexA*, *Lys1*, and *Lys2* genes were amplified using cDNA from control, HexAgs, and Lys1gs strains. Abbreviations are: HexA, β-hexosaminidase α-subunit; Lys1, lysozyme 1; gs, gene-silenced. (B) The kinetics of CHO cell destruction by control, HexAgs, and Lys1gs strains. Approximately 5×10^4^ cells of the control and HexAgs and Lys1gs strains were added to a monolayer of confluent CHO cells in a well of a 24-well plate and incubated at 35°C for the indicated times. Monolayer destruction is expressed as the percentage of destroyed CHO cells.
(TIF)

Figure S3 Localization of CPBF8AAA1-HA, CPBF8AAA2-HA, and SRR-HA to phagosomes. Trophozoites of CPBF8AAA1-HA (A), CPBF8AAA2-HA (B), and SRR-HA (C) were incubated with CellTracker Blue-stained CHO cells (*blue*) for 60 min, fixed, and reacted with anti-HA antibody (*green*). Bar, 10 µm.
(TIF)

Table S1 The list of all detected peptides from excised bands by LC-MS/MS.
(XLSX)
