# Supplementary figures and images for: Novel Transmembrane Receptor Involved in Phagosome Transport of Lysozymes and β-Hexosaminidase in the Enteric Protozoan Entamoeba histolytica
Source: PLoS Pathog. 2012 Feb 23;8(2):e1002539. doi: 10.1371/journal.ppat.1002539 (PMC3285589; doi:10.1371/journal.ppat.1002539)

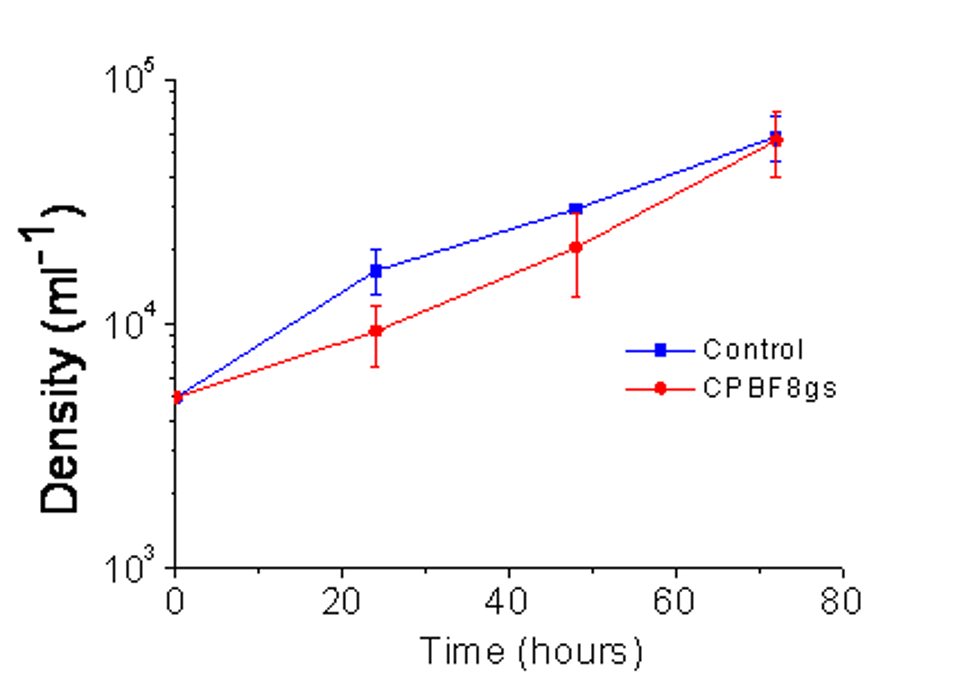

Supplement: Figure S1 — Growth kinetics of control and CPBF8gs strains. Approximately five thousands amoebae were inoculated to 6-ml culture medium. The amoebae of each strain were counted every 24 hours. Data shown are the means ± standard deviations of five biological replicates. (TIF) [file ppat.1002539.s001.tif]

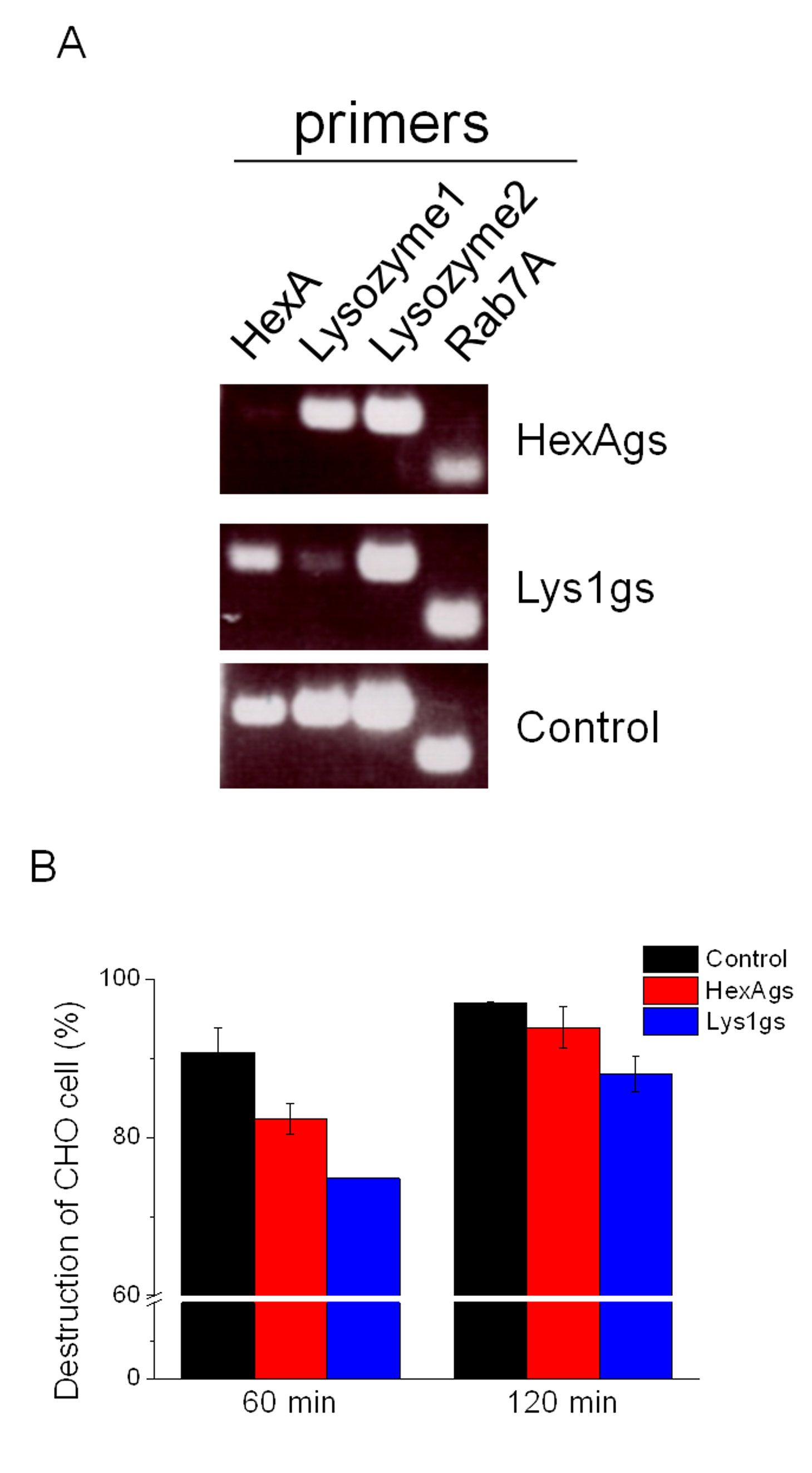

Supplement: Figure S2 — Confirmation of gene silencing in β-hexosaminidase α-subunit (HexAgs) and lysozyme 1 (Lys1gs) gene-silenced strains and their ability for the destruction of CHO monolayers. (A) Reverse transcriptase-PCR analysis. A 420-bp long partial HexA, Lys1, and Lys2 genes were amplified using cDNA from control, HexAgs, and Lys1gs strains. Abbreviations are: HexA, β-hexosaminidase α-subunit; Lys1, lysozyme 1; gs, gene-silenced. (B) The kinetics of CHO cell destruction by control, HexAgs, and Lys1gs strains. Approximately 5×10^4^ cells of the control and HexAgs and Lys1gs strains were added to a monolayer of confluent CHO cells in a well of a 24-well plate and incubated at 35°C for the indicated times. Monolayer destruction is expressed as the percentage of destroyed CHO cells. (TIF) [file ppat.1002539.s002.tif]

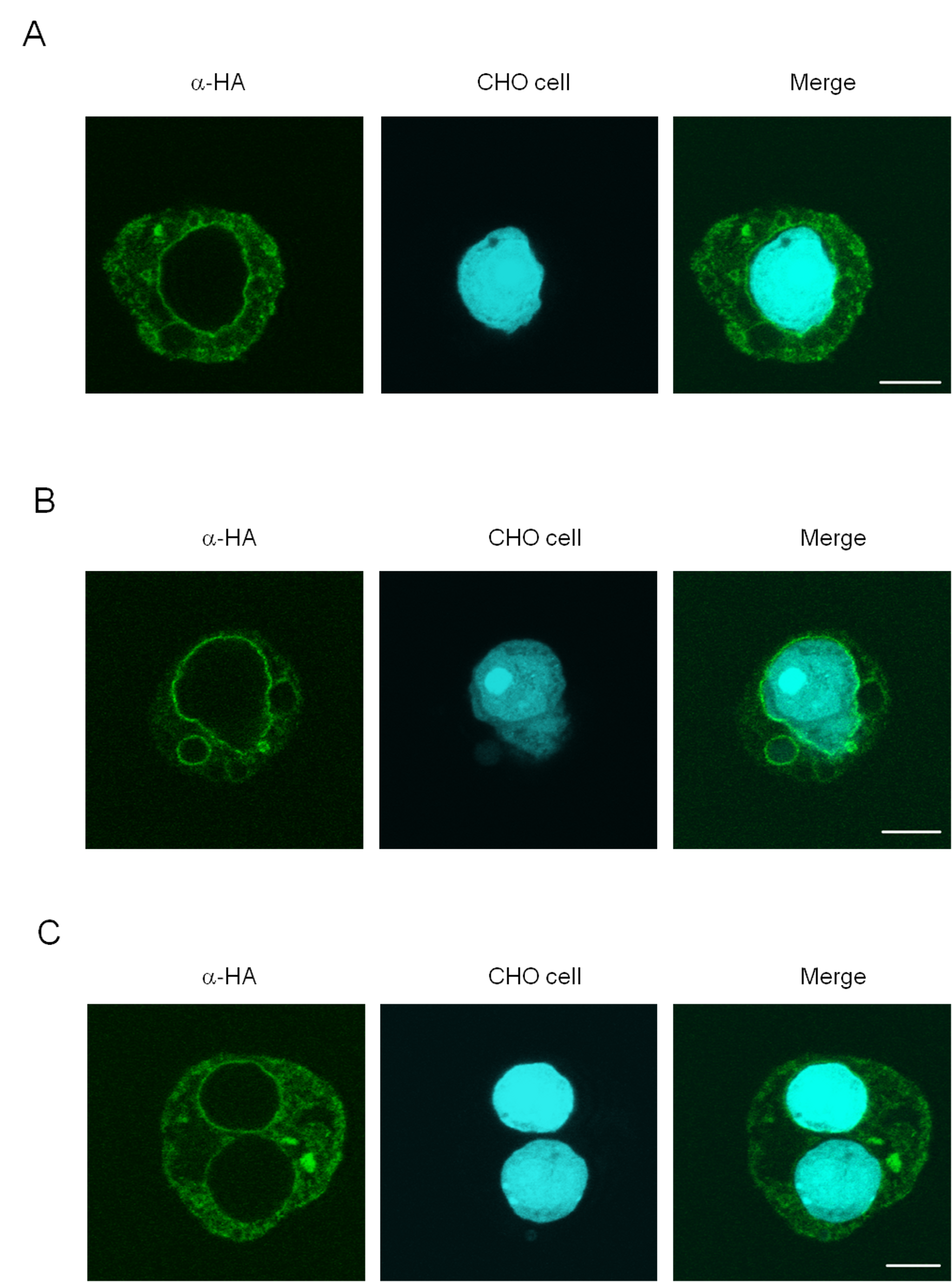

Supplement: Figure S3 — Localization of CPBF8AAA1-HA, CPBF8AAA2-HA, and SRR-HA to phagosomes. Trophozoites of CPBF8AAA1-HA (A), CPBF8AAA2-HA (B), and SRR-HA (C) were incubated with CellTracker Blue-stained CHO cells (blue) for 60 min, fixed, and reacted with anti-HA antibody (green). Bar, 10 µm. (TIF) [file ppat.1002539.s003.tif]
